# The chondrogenic response to exercise in the proximal femur of normal and mdx mice

**DOI:** 10.1186/1471-2474-11-198

**Published:** 2010-09-03

**Authors:** David J Nye, Jeffrey M Costas, Jessica B Henley, Jin-Kwang Kim, Jeffrey H Plochocki

**Affiliations:** 1 Arizona College of Osteopathic Medicine, Midwestern University, Glendale, AZ 85308, USA; 2 The Pennsylvania State University, University Park, PA 16802, USA

## Abstract

**Background:**

Submaximal exercise is used in the management of muscular dystrophy. The effects of mechanical stimulation on skeletal development are well understood, although its effects on cartilage growth have yet to be investigated in the dystrophic condition. The objective of this study was to investigate the chondrogenic response to voluntary exercise in dystrophin-deficient mice.

**Methods:**

Control and dystrophin-deficient (mdx) mice were divided into sedentary and exercise-treated groups and tested for chondral histomorphometric differences at the proximal femur.

**Results:**

Control mice ran 7 km/week further than mdx mice on average, but this difference was not statistically significant (*P *> 0.05). However, exercised control mice exhibited significantly enlarged femur head diameter, articular cartilage thickness, articular cartilage tissue area, and area of calcified cartilage relative to sedentary controls and exercised mdx mice (*P *< 0.05). No differences were found between other treatment groups.

**Conclusions:**

Mdx mice exhibit a reduced chondrogenic response to increased mechanical stimulation relative to controls. However, no significant reduction in articular dimensions was found, indicating loss of chondral tissue may not be a clinical concern with dystrophinopathy.

## Background

Genetic mutations affecting the expression of the dystrophin gene, as with Duchenne muscular dystrophy (DMD), impair cellular ability to resist muscle contractile forces and result in striated muscle cell death and fibrosis of the investing connective tissue [1,2]. Although there is no cure for muscular dystrophy, exercise has long been prescribed as a treatment modality [3,4]. Submaximal, low-intensity exercise has been shown to improve skeletal muscle performance [5], while rigorous exercise accelerates the dystrophic process [6-8]. Maintenance of muscle mass through exercise has also been shown to have musculoskeletal benefits related to gait, prolonged ambulation, and joint contracture [3,9,10]. While dystrophin deficiency does not directly affect bone and cartilage growth, the growth of these tissues is mechanically regulated and therefore indirectly affected by strains from muscle contraction [11]. Bone fractures, low bone mineral density, pelvic obliquity, and kyphoscoliosis have been attributed to the effects of muscular degeneration and joint contractures on bone growth and maintenance in dystrophin-deficient patients [12-16]. However, the precise effects of moderate exercise on articular cartilage growth with dystrophinopathy-related muscle degeneration have yet to be studied.

We examine the effects of voluntary exercise activity on the proximal femurs of juvenile dystrophin-deficient (mdx) mice. Mdx mice do not have DMD, but exhibit a similar X-linked myopathy caused by dystrophin deficiency. Mdx mice have significantly reduced skeletal myocyte diameter, numerous necrotic myocytes, and abundant fibrosis leading to significantly weaker muscle force generation relative to wild-type mice and thus serve as a useful model for testing the effects of dystrophin-related muscle weakness [17,18]. Voluntary, rather than forced, exercise is used in our study because it has been shown to help maintain muscle strength in mdx mice and is similar to the submaximal, low-intensity exercise prescribed by some physicians in the management of human dystrophinopathies [19,20].

## Methods

Twenty mice of the control strain C57BL/10ScSn (000476; Jackson Laboratory, Bar Harbor, ME) and twenty mice of the dystrophin-deficient strain C57BL/10ScSn-Dmd^mdx ^(mdx mice; 001801; Jackson Laboratory, Bar Harbor, ME) were used in the experiment. The use of animals in this study was approved by the Institutional Animal Care and Use Committee at Midwestern University and follows NIH guidelines for animal research. All mice were 7-week-old virgin females that were housed individually and provided with food and water ad libitum. Only females were used to control for potential sex differences. After a one-week acclimatization period, the mice were separated into four groups of equal size: sedentary control mice, exercise-treated control mice, sedentary mdx mice, and exercise-treated mdx mice. Exercise treatment consisted of voluntary access to a running wheel that lasted four weeks. Individual running distances were monitored using digital counters attached to each wheel. Following the four-week treatment period, the mice were sacrificed using compressed CO_2 _at the age of 11 weeks.

Femurs were immediately excised and placed in decalcifier (Surgipath, USA) for 3 days. Once decalcification was complete, the femurs were frozen in liquid nitrogen and cryosectioned at a thickness of 12 μm in the coronal plane. Sections were stained with toluidine blue to distinguish cartilage, calcified cartilage, and bone. Toluidine blue orthochromatic staining intensity of articular cartilage was observed to assess proteoglycan content of the tissue. Quantitative evaluation of toluidine blue stain is not recommended because of the variability of staining intensity and metachromasia related to decalcification, fixation, and pH of the tissue [21-24]. However, toluidine blue orthochromasia (i.e., blue appearance) is directly proportional to proteoglycan content of the tissue and thus suitable for a generalized qualitative assessment [24,25].

Histomorphometric measurements were taken on digital images captured using an Eclipse 55i microscope (Nikon Inc.). Measurements included medial-lateral femoral head diameter, cartilage thickness at midjoint, area of the calcified cartilage zone, and cartilage tissue area excluding the calcified cartilage zone. Because of differences in body mass, statistical treatment of the data consisted of a general linear model of covariance (ANCOVA) with body weight as the covariate. Statistical significance was set at *P *< 0.05.

## Results and Discussion

**Figure 1:**
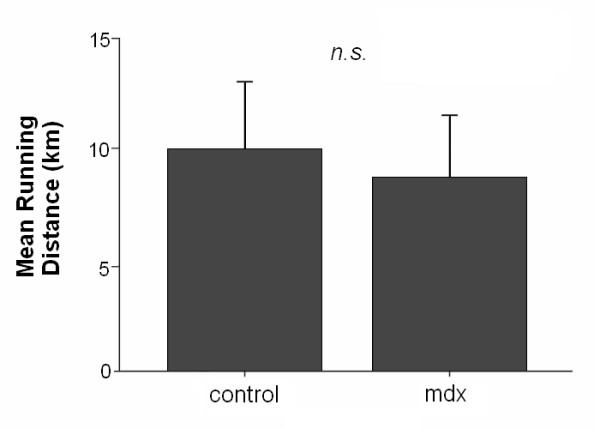
**Mean daily running distance of exercised control and exercised mdx mice**. No significant difference was found between mouse strains, although control mice ran an average of 1.01 km/day more than exercised mdx mice. Error bars depict mean ± standard deviation.

**Figure 2:**
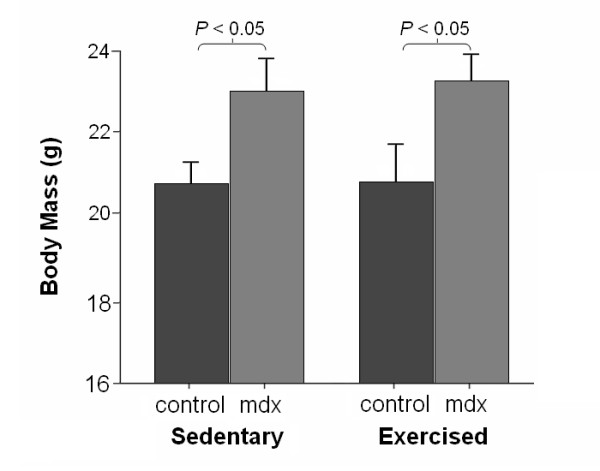
**Body mass in control and mdx by treatment groups at 11 weeks of age**. Mdx mice have significantly greater body mass than treatment-matched controls. Error bars depict mean ± standard deviation.

Average daily running distance did not differ significantly between control and mdx mice (P > 0.05, Fig. 1). On average, control mice ran 1.01 km/day more than mdx mice. One mdx mouse did not run at all and was excluded from the analysis. Mdx mice were significantly heavier than controls in both the sedentary and exercise groups (Fig. 2). However, body mass did not differ significantly between sedentary and exercised mice of the control strain or between sedentary and exercised mdx mice.

**Figure 3:**
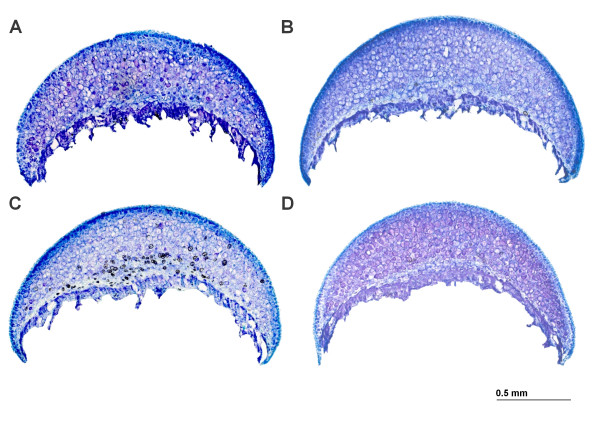
**Representative photomicrographs of femoral head articular cartilage of A) sedentary control mice, B) exercised control mice, C) sedentary mdx mice, and D) exercised mdx mice**. Exercised control mice had the largest femur head diameter, cartilage tissue area, and area of calcified cartilage in comparison to all other groups when corrected for differences in body mass. Toluidine blue, 40×.

Representative histological sections from each treatment group are shown in Fig 3. In general, the intensity of toluidine blue orthochromatic staining was greater in the control groups in comparison to mdx groups, indicating greater proteoglycan content in control mice. Statistical comparisons of histomorphometric parameters of the proximal femur corrected for body mass are displayed in Table 1. These data show that proximal femoral tissue of juvenile mdx mice is less responsive to mechanical stimulation in comparison with controls. No significant differences between sedentary and exercised mdx mice were found for any dependent variable included in the study (*P *> 0.05). However, femur head diameter, cartilage thickness, and cartilage tissue area are significantly larger in exercised controls relative to sedentary controls and exercised mdx mice (*P *> 0.05). There is an abundance of data demonstrating articular cartilage growth, bone growth, and the size of the calcification zone are sensitive to moderate exercise in healthy subjects [26-30]. *In vitro *studies confirm that mechanical loading of cartilage stimulates cell division and matrix synthesis [31,32], even in the moderate range of 10 MPa [33,34]. *In vivo*, these effects translate to elevated chondral tissue expansion under increased mechanical stimulation [28,35,36].

**Table 1:** Comparison of histomorphometric parameters of the proximal femur between treatment groups.

	Control			Mdx			Sedentary control vs. sedentary mdx	Exercise control vs. exercised mdx
			
	Sedentary	Exercised	*P*	Sedentary	Exercised	*P*	*P*	*P*
Femur head diameter (mm)	1.363 ± 0.066	1.377 ± 0.042	0.03	1.273 ± 0.112	1.320 ± 0.067	0.22	0.12	0.01
Cartilage thickness (μm)	6.081 ± 1.560	5.878 ± 0.651	0.01	5.250 ± 1.118	5.058 ± 0.872	0.70	0.46	0.01
Calcified cartilage area (μm^2^)	0.143 ± 0.041	0.206 ± 0.022	0.01	0.172 ± 0.038	0.180 ± 0.072	0.49	0.29	0.13
Cartilage Area (mm^2^)	0.909 ± 0.170	0.949 ± 0.082	0.01	0.811 ± 0.131	0.775 ± 0.095	0.88	0.09	0.01

Values are mean ± standard deviation; ANCOVA for dependent variables with body mass as a covariate

The lack of a chondrogenic response to voluntary exercise in mdx mice is a unique finding. Reduced mechanical loading and endocrinological changes may serve as possible explanations. First, it is conceivable that joint forces in mdx mice failed to provide sufficient mechanical pressure to yield significant chondral expansion. Skeletal muscle of mdx mice of similar age to the ones used in this study have been shown to contain extensive fibrosis and degenerative myocytes with diminished muscle force production [17,37,38]. While absolute force production of mdx hindlimb skeletal muscle has been reported to be similar to controls, muscle force normalized by muscle cross-sectional area is significantly reduced in mdx mice [38-40]. Thus, mdx mice are weaker for their given mass [39]. Studies have also found a significant and irreversible drop in force production during repeated eccentric muscle contraction in mdx mice that is attributed to dystrophic sarcolemma damage [41,42]. Such decreases in muscle force production accelerate fatigue, thereby reducing running endurance and speed [42,43]. For example, Hayes and Williams [44] found that mdx mice run an average of 0.5 km/hr slower than controls. Running speed also plays a large role in determining joint forces. Faster mouse running speeds increase limb joint forces, even if ground reaction force remains constant [45]. Given these reported findings, a reduction in joint forces resulting from weaker muscle force production and slower running speeds may explain the diminished chondrogenic response to voluntary running exercise in the proximal femurs of mdx mice. However, more data is needed to confirm the role of mechanical factors affecting the functional adaptation of mdx mouse limb joints.

Second, it is also possible that endocrinological factors contributed to the poor chondrogenic response to exercise in the mdx mice. Proinflammatory cytokines, such as tumor necrosis factor-alpha (TNF-α), are released during the breakdown and necrosis of dystrophic skeletal muscle myocytes [46-48]. TNF-α is a strong chemotactic agent that attracts neutrophils and macrophages to damaged tissue and can contribute to the degradation of healthy tissue. Several studies have found elevated levels of TNF-α in both skeletal muscle and blood plasma of dystrophin-deficient patients [49,50]. Circulating TNF-α in dystrophin-deficient mice could potentially affect articular cartilage where it is known to attenuate chondrocytic synthesis of proteoglycans and collagen [51-53]. Although detecting TNF-α was beyond the scope of this study, if present, the inhibitory effect on chondrocyte extracellular matrix production could explain the reduced chondrogenic response to running activity and the weak toluidine blue orthochromatic staining observed in the mdx mice relative to controls (Fig. 3). However, future studies are needed to examine proinflammatory cytokine levels in skeletal muscle, blood serum, and synovial fluid in the dystrophic condition to elucidate their relationship with chondrocyte metabolism and articular cartilage histomorphometric properties.

There is also the possibly that the inhibition of dystrophin expression in chondrocytes may affect chondrocyte proliferation and secretion of extracellular matrix, although this seems unlikely. To our knowledge, no study has identified dystrophin synthesis in chondrocytes. Additionally, other connective tissue cells, such as fibroblasts, do not produce detectable amounts of dystrophin [53,54]. Thus it remains unclear what role, if any, dystrophin plays in cartilage tissue growth and maintenance. Nonetheless, this possibility should be explored in future studies.

It should be noted that chondral tissues of the mdx mice, although not enlarged, are also not significantly reduced in size relative to sedentary controls (Table 1). Both reduced mechanical stimulation and increased levels of TNF-α can retard articular tissue growth, leading to thinner articular cartilage and smaller joints [30,55-57]. Our data suggests that chondrogenic activity in both the sedentary and exercised mdx mice aged 11 weeks is still sufficient to maintain articular tissue size comparable to sedentary controls. These findings are of interest to clinicians because, 1) low intensity exercise is sometimes used in the management of muscular dystrophy, and 2) muscular dystrophy patients lead a more sedentary lifestyle. Although the mdx mouse model does not perfectly reproduce the progression and severity of the dystrophic process observed in humans, by 6 weeks of age mdx mice exhibit significant muscle weakness and thus serve as a useful model for studying dystrophin-related muscle-skeletal tissue interactions [11]. Our findings suggest that the addition of articular tissue during postnatal growth is limited in mdx mice. However, chondral tissue area was preserved in the sedentary groups, indicating loss of chondral tissue is not a major concern with dystrophin deficiency. This may explain the lack of articular cartilage involvement in dystrophinopathies.

## Conclusions

The results of this study show mdx mice exhibit a reduced chondrogenic response to increased mechanical stimulation relative to controls. Voluntary running exercise does not significantly affect femur head diameter, cartilage thickness, cartilage tissue area, and calcified cartilage tissue area in mdx mice as it does in controls. However, articular dimensions analyzed in this study were not reduced in mdx mice in comparison with controls, suggesting loss of chondral tissue may not be a clinical concern with dystrophinopathy.

## Competing interests

The authors declare that they have no competing interests.

## Authors' contributions

DJN, MJC, JBH, and JHP contributed to the design of the experiments. JBH was primarily responsible for animal care and treatment. DJN was responsible for data collection. DJN and JHP were equally responsible for data analysis and drafting the manuscript. J-KK aided in the revision of the manuscript and interpretation of the data. All authors have read and approved the final manuscript.

## Pre-publication history

The pre-publication history for this paper can be accessed here:

http://www.biomedcentral.com/1471-2474/11/198/prepub
